# Prevalence of herbal medicine use and associated factors among pregnant women attending antenatal care at public health facilities in Hossana Town, Southern Ethiopia: facility based cross sectional study

**DOI:** 10.1186/s13690-016-0118-z

**Published:** 2016-02-15

**Authors:** Tariku Laelago, Tadele Yohannes, Fiseha Lemango

**Affiliations:** Department of health extension, Hossana College of health sciences, Hossana, Ethiopia; Department of health information technology, Hossana College of health sciences, Hossana, Ethiopia

**Keywords:** Herbal medicine, Pregnancy, Factors, Ethiopia

## Abstract

**Background:**

The use of herbal medicine has been on increase in many developing and industrialized countries. More pregnant women use herbal remedies to treat pregnancy related problems due to cost-effectiveness of therapy and easy access of these products. We sought to assess the prevalence of herbal medicine use and associated factors among pregnant women attending antenatal clinics of public health facilities.

**Methods:**

Facility based cross sectional study was conducted among 363 pregnant women attending antenatal clinics from May to June 2015 at public health facilities in Hossana town, Hadiya zone, Southern Ethiopia. Pretested structured questionnaire was used to collect data from each study subject. Bivariate logistic regression analysis was used to see significance of association between the outcome and independent variables. Odds ratios at 95 % CI were computed to measure the strength of the association between the outcome and the independent variables. *P*-value <0.05 was considered as a statistically significant in multivariate analysis.

**Result:**

Two hundred fifty eight (73.1 %) of pregnant women used herbal medicine during current pregnancy . The herbal medicines commonly taken during current pregnancy were ginger (55.8 %), garlic (69.8 %), eucalyptus (11.6 %), tenaadam (rutachalenssis) (26.4 %), damakesse (ocimumlamiifolium) (22.8 %), feto (3.5 %) and omore (3.1 %). Being students (AOR: (5.68, 95 % CI: (1.53, 21.13), second trimester of pregnancy (AOR: 0.22, 95 % CI: (0.08, 0.76), sufficient knowledge on herbal medicine (AOR: 0.37, 95 % CI: (0.19, 0.79), no formal education (AOR: 4.41, 95 % CI: (1.11, 17.56), primary education (AOR: 4.15, 95 % CI: (1.51, 11.45) and secondary education (AOR: 2.55, 95 % CI: (1.08,6.03) were significantly associated with herbal medicine use.

**Conclusion:**

The findings of this study showed that herbal medicine use during pregnancy is a common experience. Commonly used herbal medicines during current pregnancy were garlic, ginger, tenaadam, damakasse and eucalyptus. Educational status, occupation, knowledge on herbal medicine and second trimester of pregnancy were the major factors affecting use of herbal medicine. Health education about the effects of herbal medicine on pregnancy should be given during antenatal care sessions and through media. Health care providers, especially those that are involved in antenatal care should aware of evidence regarding potential benefits or harm of herbal medicinal agents when used by pregnant women.

## Background

The World Health Organization (WHO) defines traditional medicine as health practices, approaches, knowledge and beliefs incorporating plant, animal and mineral based medicines, spiritual therapies, manual techniques and exercises, applied singularly or in combination to treat, diagnose and prevent illnesses and maintain well-being [1].

Herbal medicines, which is part of traditional medicines are defined as plant-derived material or preparations perceived to have therapeutic benefits; they often contain raw or processed ingredients from one or more plants [2]. Herbal medicines include herbs, herbal materials, herbal preparations, and finished herbal products that contain parts of plants or other plant materials as active ingredients [3]. It is known that between 65 and 80 % of the world’s population use herbal medicines as their primary form of health care [4]. Patients who are likely to be at risk from adverse effects of herbal medicines include those who are already prone to difficulties from regularly prescribed medications namely foetus, infants and older children, the elderly, as well as pregnant and lactating women [5].

In Asian countries such as China, traditional medicine accounts for around 40 % of all health care delivered. In Africa up to 80 % of the population uses traditional medicine to help meet their health care needs [6]. The use of herbal medicine in Africa is associated with a lower level of education, use before the index pregnancy, age, low economic status and trimester of pregnancy [7, 8].

Up to 80 % of Ethiopian population use traditional medicine. The reasons for traditional medicines uses in Ethiopia are the cultural acceptability of healers and local pharmacopeia’s, the relatively low cost of traditional medicine and difficult access to modern health facilities [9]. Study conducted on use of herbal medicine among pregnant women on one hospital of Ethiopia showed that 69.84 % herbs were used during the first trimester and 4.76 % of women used herbs in all trimesters. Commonly used herbs were ginger (44.36 %), garlic (37.32 %), eucalyptus (9.15 %) and tenaadam (9.15 %) [10].

Herbal medicines used by pregnant women were self-prepared mostly by mixing different parts of plants, while some took prepared and pre-packaged herbal medicinal products. One of the major reasons cited by pregnant women for taking herbal remedies included higher efficacy of herbal medicines when compared with conventional medicines, safety in pregnancy (because they are natural products), beliefs concerning cultural heritage of herbal medicines and comparatively low cost of herbal medicines [11].

Studies have also reported different characteristics of women, which makes them more likely to take herbal medicine in pregnancy. These included being older, married, primiparous, having tertiary education, being less educated and severity of nausea and vomiting [12–14].

Having recognized the significance of traditional medicine more readily, greater attention has been paid by governments of many developing countries in recent years to promote the widespread application of the practice in health care. This has given a new impetus to relevant research, investment and design of programs in the area in many countries [15].

The study of herbal medicine use related to maternal health, a public health priority in many African countries including Ethiopia, has been limited. Therefore, the objective of this study was to assess the prevalence of herbal medicine use and associated factors among pregnant women attending antenatal care clinics at public health facilities in Hossana town.

## Methods

### Study area

This study was conducted in Hossana town, the capital of Hadiya Zone, SNNPR of Ethiopia. The town is situated 232 Km Southwest of Addis Ababa and 194kms northwest of the regional capital Hawassa. The town has one zonal hospital, three public health centres and 25 clinics of all types. This study was conducted on antenatal clinics of the hospital and the health centres.

In Ethiopia antenatal care is given based on focused antenatal care approach. Focused antenatal care with four visits per pregnancy is mainstreamed at all service delivery levels. It is given in health posts, health Centres, hospitals and clinics.

### Study design and period

Facility based cross sectional study was conducted among 363 pregnant women from May to June 2015.

### Study participants

The source population of the study was the excepted number of pregnant women (*n* = 3984) in the town in 2014. The study population was pregnant women who were attending the antenatal clinics of the public health facilities. Women who were not mentally and physically capable of being interviewed were excluded.

### Sampling

The estimated sample size was 363 pregnant women and it was selected by systematic sampling technique.

### Data collection and measurements

Pretested structured questionnaire was used to collect data from each study subject. Majority of the questions were adopted from related literature with slight modification made in line with the objective of this particular study and to fit to the local context. Data collected on socio-demographic factors includes age, marital status, educational level, monthly income of house hold, occupation and religion of respondents.

On behavioural factors, knowledge and attitude toward herbal medicine use response was sought. Environmental factors that included in study were access to health facility, sources of information and sources to obtain herbal medicine. Knowledge on common herbal medicine use, complication of herbal medicine use to pregnant mother and foetus were obtained.

Data collected on pregnancy related factors includes parity, gravidity, stages of pregnancy and current pregnancy status. Responses were also sought on use of herbs during pregnancy, indication to use, routes and untoward effects of post administration of herbal medicines.

The questionnaire was first prepared in English, translated into Amharic and then re-translated back to English to check for its consistency.

Four nurses and two public health masters were recruited as interviewers and supervisors respectively. Data collectors were trained for 2 days on interviewing techniques, purpose of the study and ethical aspects. Pre-test was carried out on one health facility which was not included in the actual study. Based on the result, data collectors were reoriented and the questionnaire was modified as necessary. The principal investigators and supervisors made a day to day on site supervision during the whole period of data collection.

The women were considered as herbal medicines users if they took the herbal medicines through oral, intra-vaginal or topical routes. Other preparations that are consumed as nutriments and within routine meal preparation such as food additives were excluded.

The income for study participant was calculated in Ethiopian Birr. The individual respondents income was compared with the mean (≥ mean or < mean).

Knowledge was measured using 10 items prepared to assess it. Respondents were asked knowledge related questions and right answer was given a value of 1 and for those incorrect answers a value of 0 was given. Then, total score was computed by summing up all the items together. The respondents score was dichotomized as sufficient knowledge or insufficient knowledge.

- Sufficient Knowledge ≥ overall mean
- Insufficient knowledge < overall mean

Attitude was measured using seven items on five points Likert scale ranged from strongly disagree to strongly agree. After computing the mean of all respondents’ responses, the mean score of each respondent was dichotomized as have positive attitude or negative attitude.

- Positive attitude ≥ mean
- Negative attitude < mean

### Data processing and analysis

Data were entered into Epi-Data version 3.1 [16], then, it was exported into SPSS version 16.0 statistical software for analysis [17]. Different frequency tables and descriptive summaries were used to describe the study variables. Bivariate logistic regression analysis was used to see significance of association between the outcome and independent variables. Variables with *p*-value <0.05 in bivariate analysis were transferred to multivariate logistic regression. Odds ratios at 95 % CI were computed to measure the strength of the association between the outcome and the explanatory variables. *P*-values less than 0.05 were considered as statistical significant in the multivariate analysis.

### Ethical considerations

Prior to data collection, appropriate ethical clearance was taken from ethical clearance committee of Hossana College of Health Sciences. The study included patients that had given informed consent before data collection. In order to establish anonymous linkage, only the codes, not the names of the respondents, were registered on the questionnaire. During the training of data collectors and supervisor ethical issues was addressed as important component of the research.

## Results

### Socio-demographic characteristics

Of the 363 pregnant women attending the antenatal care clinic, 353 (97 %) gave an informed consent and were included in the study. The study included pregnant women irrespective of trimester. Majority of the respondents 344 (97.5 %) were married. Concerning educational level; 129 (36.5 %) of the respondents attended primary education (grade 1–8) and 129 (36.5 %) respondents’ educational level was secondary education (grade 9–12). Based on the finding minimum, mean and maximum ages of the respondents were 16, 25.4, and 40 years respectively with SD of 4.1. The mean family income of the respondents was 2116.22 Ethiopian birr per month. The minimum and the maximum family income per month was 200 and 9,900 ETB respectively. One hundred eight seven (53.0 %) of the pregnant women were housewives. Majority of respondents 238 (67.4 %) were protestant religion followers (Table 1).

**Table 1:** Socio-demographic characteristics of the respondents, Hossana, June, 2015 (*N* = 353)

Variables	Total (N and %)
Marital status(*n* = 353)	
Married	344 (97.5 %)
Single	4 (1.1 %)
Divorced	5 (1.4 %)
Occupation(*n* = 353)	
Housewives	187 (53.0 %)
Students	16 (4.5 %)
Privately owned business	61 (17.3 %)
Government employee	62 (17.6 %)
Non-governmental employee	15 (4.2 %)
Others*	12 (3.4 %)
Religion(*n* = 353)	
Protestants	238 (67.4 %)
Orthodox	81 (22.9 %)
Muslims	22 (6.2 %)
Catholics	12 (3.4 %)
Educational Status(*n* = 353)	
No formal education	35 (9.9 %)
primary(1–8)	129 (36.5 %)
Secondary (9–12)	129 (36.5 %)
More than secondary education	60 (17.0 %)
Average monthly income (*n* = 340)	
<2116.2 ETB	209 (61.5 %)
≥2116.2 ETB	131 (38.5 %)
Current pregnancy status(*n* = 353)	
Planned	273 (77.3 %)
Unplanned	80 (22.7 %)
Gravidity (*n* = 353)	
1–2 pregnancy	252 (71.4 %)
3–4 pregnancy	74 (21 %)
>4 pregnancy	27 (7.6 %)
Parity(*n* = 197)	
1–2 children	150 (76.1 %)
3–4 children	36 (18.3 %)
>4 children	11 (5.6 %)
Trimester of pregnancy(*n* = 353)	
First trimester	17 (4.8 %)
Second trimester	147 (41.6 %)
Third trimester	189 (53.5 %)

*Others: preachers, daily labourers

### Prevalence of herbal medicine use

Of the respondents, 258 (73.1 %) with 95 % CI :( 68.1–78.1) used herbal medicine during current pregnancy. From those who took herbal preparations during current pregnancy, 189 (53.5 %) used in the third trimester (Table 2).

**Table 2:** Utilization of herbal medicine among the respondents, Hossana, June, 2015

Variables	Total (N and %)
Herbal medicine use during current pregnancy (*n* = 353)	
Yes	258 (73.1 %)
No	95 (26.9 %)
Reasons to use herbal medicine over other medicine (*n* = 258)	
Safe in pregnancy	42 (16.3 %)
Cheap	24 (9.3 %)
Accessible	106 (41.1 %)
Effective	101 (39.1 %)
Routes through which herbal medicines were used (*n* = 258)	
Oral	219 (84.9 %)
Topical	26 (10.1)
Others*	13 (5 %)
Experienced untoward effect on post administration (*n* = 258)	
Yes	21 (8.1 %
No	237 (91.9 %)
Untoward effects on post administration (*n* = 21)**	
Burning sensation	9 (43 %)
Vomiting	7 (33.3 %)
Dizziness	4 (19.0 %)
Malaise	4 (19.0 %)
Headache	3 (14.3)
Others***	5 (23.8 %)

*intra-vaginal, intranasal

**More than one response

***diarrhoea, abdominal pain, rashes

**Fig. 1:**
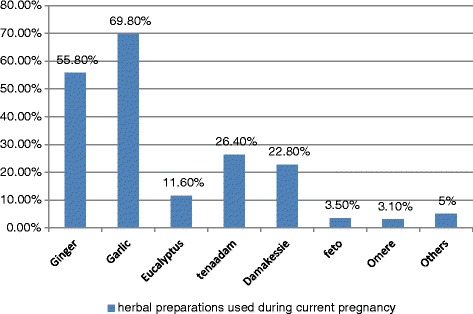
Herbal preparations used during current pregnancy, Hossana, June, 2015

The herbal preparations that had taken by the respondents during the current pregnancy were ginger 144 (55.8 %), garlic 180 (69.8 %), eucalyptus 30 (11.6 %), tenaadam 68 (26.4 %), damakesse 59 (22.8 %), feto 9 (3.5 %), omore 8 (3.1 %) and others (anamura, barewa, duba fire, limich, tosign (thymus serrulatus) 13 (5.0 %) (Fig. 1).

Indications for using the herbal medicine during pregnancy were nausea 82 (31.8 %), vomiting 42 (16.3 %), abdominal pain 64 (24.8 %), cold 185 (71.7 %), malaria 8 (3.1 %) and others (amoebiasis, fever, taeniasis, typhoid fever, tonsillitis, diarrhoea) 55 (21.3 %).

### Knowledge and attitude of pregnant women on herbal medicine use

From the participants included in this study, 92.1 % of the respondents heard about herbal medicine from different sources. With respect to knowledge on herbal medicine, 80.7 % of the respondents had sufficient knowledge on herbal medicine. From the respondents, 169 (47.9 %), 114 (32.3 %), 105 (29.7 %) raised abortion, excessive uterine contraction, uterine rupture respectively as possible complications of herbal medicine use.

Out of 353 respondents, 184 (52.1 %) had positive attitude and 169 (47.9 %) had negative attitude towards herbal medicine use (Table 3).

**Table 3:** Knowledge and attitude of pregnant women on herbal medicine use, Hossana, June, 2015

Variables	Total (N and %), *N* =353
Knowledge on herbal medicine	
Sufficient knowledge	285 (80.7 %)
Insufficient knowledge	68 (19.3 %)
Heard about herbal medicine	
Yes	323 (92.1 %)
No	28 (7.9 %)
Types of herbal medicine respondents knew*	
Garlic (Allium sativum)	270 (76.5 %)
Ginger (zingiberofficinale)	253 (71.7 %)
Eucalyptus	159 (45 %)
Tenaadam (Rutachalenssis)	233 (66 %)
Damakesse (ocimumlamiifolium)	81 (25 %)
Omore	10 (2.8 %)
Others**	31 (8.5 %)
Possible complications replied by the respondents*	
Abortion	169 (47.9 %)
Excessive uterine contraction	114 (32.3 %)
Uterine rupture	105 (29.7 %)
Attitude	
Positive	184 (52.1 %)
Negative	169 (47.9 %)

*More than one possible answers were used

**berawa, feto, duba fire, tosign, limich

### Pregnancy related factors

Concerning current pregnancy status, around three fourth 273 (77.3 %) were planned pregnancy and the rest 80 (22.7 %) were unplanned. Out of the 353 respondents, 150 (76.1 %) of women had 1–2 children. From the pregnant women included in this study, 71.4 % of women became pregnant 1–2 times. Regarding trimester, around half 189 (53.5 %) of the respondents were in the third trimester (Table 1).

### Environmental factors

Out of the respondents, 214 (60.6 %) had access to health facility in less than five kilometres and only few 28 (7.8 %) walk more than 10 km to get nearby health facility. Concerning source of information, nearly half 159 (49.2 %) of the respondents obtained information about herbal medicine from parents/relatives. From those women who used herbal medicine during pregnancy, 105 (41.5 %) got from market places and 76 (29.5 %) prepared by themselves (Table 4).

**Table 4:** Environmental factors among pregnant women, Hossana, June, 2015

Variables	Total (N and %)
Access to health facility(*n* = 353)	
≤5 km	214 (60.6 %)
5–10 km	111 (31.4 %)
>10 km	28 (7.8 %)
Source of information (*n* = 323)*	
Parents/relatives	159 (49.2 %)
Health professionals	16 (5 %)
Neighbours	109 (33.7 %)
Herbalist	28 (8.7 %)
Others**	41 (12.7 %)
Sources to obtain herbal medicine (*n* = 258)*	
Self-preparation	76 (29.5 %)
Traditional herbalist	30 (11.6 %)
Work places	18 (7 %)
Market places	107 (41.5 %)
Neighbours	67 (26 %)
Others**	55 (21.3 %)

*More than one possible answer was used

**Friends, mass media

### Factors associated with herbal medicine use

Selected variables that were significantly associated at the bivariate analysis were further examined in the in multivariate logistic regression to see their relative effects on the use of herbal medicine.

Result of bivariate analysis showed that educational status (*p* < 0.001), occupation (*p* < 0.001), average monthly income (*p* < 0.05), knowledge (*p* < 0.05), attitude of the respondents towards herbal medicine (*p* ≤ 0.001) and trimester of pregnancy (*p* < 0.05) as candidates for multivariate analysis at *p*-value <0.05 (Table 5).

**Table 5:** Factors associated with the utilization of herbal medicine, Hossana, June, 2015

Variable	Use of herbal medicine N (%)			
	No	Yes	COR at 95 % CI	AOR at 95 % CI
Educational status				
No formal education	6 (17.1 %)	29 (82.9 %)	6.77 (2.445,18.726)*	4.41 (1.107,17.556)***
Primary	18 (14 %)	111 (86 %)	8.63 (4.223,17.651)*	4.15 (1.506,11.447)**
Secondary	36 (27.9 %)	93 (72.1 %)	3.62 (1.904,6.869)*	2.55 (1.083,6.025)***
More than secondary	35 (58.3 %)	25 (41.7 %)	1	1
Occupation				
Housewife	28 (15.0 %)	159 (85.0 %)	1	1
Students	7 (43.8 %)	9 (56.2 %)	5.68 (1.709,18.868)***	5.68 (1.528,21.129)***
Privately owned business	16 (26.2 %)	45 (73.8 %)	1.29 (0.286,5.774)	1.26 (0.231,6.858)
Governmental employee	31 (50 %)	31 (50 %)	2..81 (.792,9.987)	2.55 (0.640,10.164)
Non-governmental employee	7 (46.7 %)	8 (53.3 %)	1.00 (0.290,3.443)	1.63 (0.376,7.068)
Others	6 (50 %)	6 (50 %)	1.14 (0.250,5.224)	1.97 (0.356,11.067)
Average monthly income				
<2116.22	47 (35.9 %)	84 (64.1 %)	1	1
≥2116.22	43 (20.6 %)	166 (79.4 %)	2.16 (1.323,3.525)**	1.07 (0.579,1.9770
Knowledge on use of herbal medicine				
Insufficient knowledge	26 (38.2 %)	42 (61.8 %)	1	1
Sufficient knowledge	69 (24.2 %)	216 (75.8 %)	0.516 (0.295,0.903)***	0.37 (0.189,0.789)**
Attitude towards use of herbal medicine				
Negative attitude	32 (18.9 %)	137 (81.1 %)	1	1
Positive attitude	63 (34.2 %)	121 (65.8 %)	2.23 (1.365,3.641)*	1.67 (0.941,2.972)
Trimester of pregnancy				
First trimester	9 (52.9 %)	8 (47.1 %)	1	1
Second trimester	44 (29.9 %)	103 (70.1 %)	0.25 (0.092,0.699)**	0.22 (0.078,0.762)***
Third trimester	42 (22.2 %)	147 (77.8 %)	0.67 (0.409,1.094)	0.74 (0.414,1.312)

Significant at *p* ≤ 0.001*; *p* < 0.005**; *p* < 0.05***

In multivariate logistic regression analysis educational status, occupation of respondents, knowledge on use of herbal medicine and trimester of pregnancy were significantly associated with herbal medicine use (Table 5).

Pregnant women who had no formal education were 4.4 times higher to use herbal medicine as compared to those women who attend more than secondary education(AOR: 4.41, 95 % CI: (1.107, 17.556)). Pregnant who had primary education were 4.1 times higher to use herbal medicine as compared to those who attended more than secondary education (AOR: 4.15, 95 % CI: (1.506, 11.447)) and pregnant women who attended secondary education were 2.6 folds higher to use herbal medicine during the current pregnancy as compared to those who do attended more than secondary (AOR: 2.55, 95 % CI: (1.083, 6.025)).

The odds of using herbal medicine during pregnancy was 5.7 folds higher in students as compared to housewives (AOR: (5.68, 95 % CI: (1.528, 21.129)).

Respondents who had sufficient knowledge on herbal medicine use were 63 % less likely to use herbal medicine during pregnancy as those who had insufficient knowledge on herbal medicine (AOR: 0.37, 95 % CI: (0.189, 0.789). Respondents who were in the second trimester were 78 % less likely to use herbal medicine as compared to those who were in the first trimester (AOR: 0.22, 95 % CI: (0.078, 0.762)).

## Discussion

In this study an attempt has been made to assess the prevalence of herbal medicine use and associated factors among pregnant women attending antenatal clinics.

The findings of this study revealed that 73.1 % (95 % CI :( 68.1 %, 78.1 %)) used herbal medicine during current pregnancy, this is higher than the findings of study conducted at Nekemte hospital, Western Ethiopia, which reported as 50.4 % [10]. It was also around 2 folds higher than the survey report at Ulleval University hospital in Oslo in 2004, which showed herbal medicine use as 36 % [8, 18]. This may be due to accessibility, affordability and/or safety of the herbs at different districts of the country. It also may be due to difference in culture, tradition and ready access to health care.

This study showed commonly used herbal medicines during pregnancy as garlic (68.80 %), ginger (55.80 %), tenaadam (26.4 %), damakasse (22.8 %) and eucalyptus (11.6 %) which is in line with the findings of study conducted at Nekemte hospital, Western Ethiopia in which ginger (44.36 %), garlic (37.32 %), eucalyptus (9.15 %) and tenaadam (9.15 %) but study conducted in Northern Nigeria showed only garlic and ginger as commonly used herbal medicine during pregnancy[8, 10]. This may due to the accessibility of herbs. There is also difference in geographical location hence this will affect the plants commonly grow in the countries.

The likelihood of herbal medicine use was 2.6, 4.1 and 4.4 folds higher among women who attended secondary, primary and no formal education respectively as compared to those who did attend more than secondary education. This finding is in line with the results of a study conducted on use of herbal medicine among pregnant women on antenatal care at Nekemte hospital, Western Ethiopia which showed education has significant association with use of herbal medicine [10]. Study conducted at Mbeya referral hospital and Northern Nigeria among women attending antenatal clinic showed the use of herbal medicines during pregnancy was associated with low education level of pregnant women [8, 19]. The higher educational attainment in this study group may explain the lower prevalence of herbal medicine use.

The odds of using herbal medicine during pregnancy were 5.7 folds higher in students as compared to housewives. This is similar with the findings of results of a multinational study in which women who were currently students were more likely to use herbal medicines than other women [20].

Respondents who had sufficient knowledge on herbal medicine use were 63 % less likely to use herbal medicine during pregnancy as those who had insufficient knowledge on herbal medicine. This finding is in line with the results of a study done in tertiary hospital in Northern Nigeria which revealed that knowledge on herbal medicine had significant association with its use [18].

Respondents who were in the second trimester were less likely to use herbal medicine as compared to those who were in the first trimester but herbal medicine use have no association with third trimester pregnancy. The findings were in line with the study done in Nigeria which showed that, herbal medicine use by pregnant women had a statistically significant association with use in first and second trimesters of pregnancy but not with third trimester [20]. These findings raise concerns about fetal safety because this is a critical period of fetal organogenesis and maturation [21].

### Strength and limitation of the study

The high response rate (97 %) and exploring some herbs that were not studied previously are strength of the study. There are limitations to this study. Like all self-reported exposure assessments, under reporting is very likely. As it is cross-sectional, it fails to show seasonal variability in the use of herbal medicine.

## Conclusion

The findings of this study showed that herbal medicine use during pregnancy is a common experience. Almost three out of four women used herbal medicine during current pregnancy. Commonly used herbal medicines during current pregnancy were garlic, ginger, tenaadam, damakasse and eucalyptus. Educational status, occupation of the respondents, knowledge on herbal medicine and trimester of pregnancy were the major factors affecting use of herbal medicine.

Health education about the effects of herbal medicine on pregnancy should be given during ANC sessions and through media. Health care providers, especially those that are involved in antenatal care should be aware of evidence regarding potential benefits or harm of herbal medicinal agents when used by pregnant women. This study recommends a detailed study on commonly used herbs to establish the efficacy and safety of these herbs to ensure the well-being of the mother and foetus by concerned bodies.

## Abbreviations

ANC: Antenatal care
AOR: Adjusted odd ratio
CI: Confidence interval
ETB: Ethiopian birr
KM: kilometre
SD: Standard deviation
SNNPR: South Nation’s Nationalities Peoples Region
